# Polyphenols-Rich Extract of *Calotropis procera* Alone and in Combination with Trichoderma Culture Filtrate for Biocontrol of Cantaloupe Wilt and Root Rot Fungi

**DOI:** 10.3390/molecules29010139

**Published:** 2023-12-26

**Authors:** Ashraf M. Nofal, Ragaa A. Hamouda, Amira Rizk, Mohamed Abd El-Rahman, Adel K. Takla, Hoda Galal, Mashael Daghash Alqahtani, Basmah M. Alharbi, Amr Elkelish, Sabery Shaheen

**Affiliations:** 1 Sustainable Development Department, Environmental Studies and Research Institute, University of Sadat City, Sadat City 32897, Egypt; ashraf.nofal@esri.usc.edu.eg (A.M.N.); melsabagh495@gmail.com (M.A.E.-R.);; 2 Department of Biology, College of Sciences and Arts at Khulis, University of Jeddah, Jeddah 21959, Saudi Arabia; 3 Department of Microbial Biotechnology, Genetic Engineering and Biotechnology Research Institute (GEBRI), University of Sadat City, Sadat City 32897, Egypt; 4 Food Science and Technology Department, Faculty of Agriculture, Tanta University, Tanta City 31527, Egypt; amira.rizk@agr.tanta.edu.eg; 5 Pomology, Evaluation of Natural Resources Department, Environmental Studies and Research Institute, University of Sadat City, Sadat City 32897, Egypt; hoda.galal@esri.usc.edu.eg; 6 Department of Biology, College of Science, Princess Nourah bint Abdulrahman University, P.O. Box 84428, Riyadh 11671, Saudi Arabia; mdalqahtani@pnu.edu.sa; 7 Biology Department, Faculty of Science, University of Tabuk, Tabuk 71491, Saudi Arabia; b.alharbi@ut.edu.sa; 8 Department of Biology, College of Science, Imam Muhammad bin Saud Islamic University (IMSIU), Riyadh 11623, Saudi Arabia; amr.elkelish@science.suez.edu.eg; 9 Department of Botany and Microbiology, Faculty of Science, Suez Canal University, Ismailia 41522, Egypt

**Keywords:** root rot, wilt, bio agents, *Trichoderma* sp., plant extract, cantaloupe

## Abstract

Fungal diseases have always been a major problem for cantaloupe crops; however, synthetic fungicides are hazardous to humans and the environment. Consequently, a feasible alternative to fungicides without side effects could be by using bio agents and naturally occurring plants with antibacterial potential. This study has achieved a novel procedure for managing wilt and root rot diseases by potentially using *Trichoderma* sp. culture filtrates in consortium with plant extract of *Calotropis procera*, *Rhizoctonia solani*, *Fusarium oxysporum*, and *Pythium ultimum*, which were isolated from infected cantaloupe roots with identified root rot symptoms. The antagonistic activity of four *Trichoderma* isolates and analysis of antibiotics and filtrate enzymes of the most active *Trichoderma* isolate were determined as well as phytochemical analysis of *C. procera* plant extract using HPLC-UV. The obtained results showed that all *Trichoderma* isolates considerably lowered the radial growth of *P. ultimum*, *R. solani*, and *F. oxysporum* in varying degrees. The scanning electron micrographs illustrate the mycoparasitic nature of *Trichoderma* sp. on *F. oxysporum*. The phytochemical analysis of *C. procera* indicated that phenolic contents were the major compounds found in extracts, such as vanillin (46.79%), chlorogenic acid (30.24%), gallic acid (8.06%), and daidzein (3.45%) but including only a low amount of the flavonoid compounds rutin, naringenin, and hesperetin. The Pot experiment’s findings showed that cantaloupe was best protected against wilting and root rot diseases when it was treated with both *Trichoderma* sp. culture filtrates (10%) and *C. procera* extract of (15 mg/mL), both alone and in combination. This study demonstrates that the application of bio agent *Trichoderma* spp. filtrate with *C. procera phenol* extract appears useful for controlling wilting and root rot disease in cantaloupe. This innovative approach could be used as an alternative to chemical fungicide for the control of wilting and rot root diseases.

## 1. Introduction

Cantaloupe (*Cucumis melo* L.) belongs to the Cucurbitaceae family, which is one of the world’s most well-known fruit crops and is a great source of nutrients such as cucurbitacin, lithium, and zinc [1]. Cantaloupe cultivated in Egypt can be exported to the Arabian and Gulf markets [2]. One cup of fresh cantaloupe contains 144 calories and 6% of the daily fiber requirement, and it also contains 100% of the recommended value of vitamin C and vitamin A. It provides about 12% of the daily potassium requirement and also has high levels of vitamins and minerals such as folic acid, calcium, copper, zinc, and iron [3].

One of the main causes of the direct depletion of agricultural natural resources is plant diseases [4]. These cause 10–20% annual losses in global food production, which has a negative influence on the food supply and results in a billion-dollar financial shortfall [5]. Fungi are the most dangerous plant pathogens among soil-borne diseases, e.g., *Rhizoctonia solani*, *Fusarium oxysporum*, and *Pythium ultimum*. These three soil-borne fungi have been implicated in the root rot and wilt diseases of cantaloupe in fields. Plant diseases have been successfully prevented by using chemical control, but overuse has accelerated the emergence of infections that are fungicide-resistant. In addition, chemical application upsets the ecological equilibrium of soil microorganisms [4]. However, because fungal conidia persist for a long period, and chemical residues are dangerous to human health, employing fungicides to combat Fusarium wilt is unsuccessful [6]. Due to its variety of sources, bio-control is safe for people and animals and is ecologically sustainable [7].

*Trichoderma* fungus, an antagonist of phytopathogenic fungi, has been employed in 90% of biological controls for plant disease [4]. Because of its capacity to fight infections and perform a variety of functions, including cell wall disintegration, hyphal growth, and antagonist activity versus phytopathogens, the genus *Trichoderma* serves as a biocontrol agent [8]. Root rot and wilt diseases are caused by the pathogenic fungi *Rhizoctonia solani*, *Fusarium oxysporum*, and *Pythium ultimum*. *Trichoderma* spp. isolated from soil have demonstrated significant antifungal activities [9]. *Trichoderma* fungi are well-known producers of numerous extracellular enzymes, including cellulases, endochitinases, N-acetyl-β-galactosidases, β-1,3- and β-1,6-glucanases, proteases, and xylanases [10]. The enzymatic mixtures (xylanase, cellulase, and glucanase) produced by *Trichoderma* exhibited the highest concentration of fibrolytic enzymes and were amended to industrial feed to test their ability to hydrolyze insoluble fibers [11]. The antagonistic *Trichoderma* produces extracellular hydrolases including chitinase and glucanase, which directly attack pathogens to dissolve the cell walls of phytopathogens [12]. By using antagonistic nonpathogenic microorganisms, biological control is a good alternative way to reduce Fusarium wilt and root rot infection to limit the negative impacts in many crops [13].

Many physiologically active substances are found in plants and are used as natural substitutes for manufactured chemicals. Natural fungicides are organic bioactive substances [14]. The presence of terpenes, flavonoids, phenolics, phytosterols, and polyketides has a significant impact on the effectiveness of natural fungicides made from plant extracts [15]. Phenols, flavonoids, and tannins possess anti-radical and antioxidant characteristics and also directly inhibit the growth of some types of fungi [16]. Sodom’s apple is the common name for *Calotropis procera* (Aiton) W.T. Aiton (family Apocynaceae). It is a plant widely spread worldwide, especially in arid and semi-arid regions [17]. *Calotropis procera* extracts have been shown to have several pharmacological effects, including anticancer, anti-inflammatory, antidiabetic, antioxidant, and antibacterial [18]. Numerous investigations have determined that this plant contains some metabolites, including flavonoids, tannins, terpenoids, saponins, alkaloids, and steroids, because of its significant biological effects. These phytoconstituents serve as anti-inflammatory, anti-diarrheal, antiviral, antioxidant, and antibacterial substances, among other medicinal functions [19].

Thus, the objective of this study is to assess the antifungal efficacy of *Trichoderma* isolate (*Trichoderma* spp.) culture filtrates and a phenolic extract of *Calotropis procera* in preventing wilting and root rot diseases in cantaloupe plants as caused by *Pythium ultimum*, *Fusarium oxysporum*, and *Rhizoctonia solani*, both in vitro and in vivo.

## 2. Results and Discussion

### 2.1. In Vitro Experiments

#### 2.1.1. Screening for Antagonistic Potential of *Trichoderma* Isolates against Tested Phytopathogenic Fungi (Dual-Culture Experiments)

**Figure 1:**
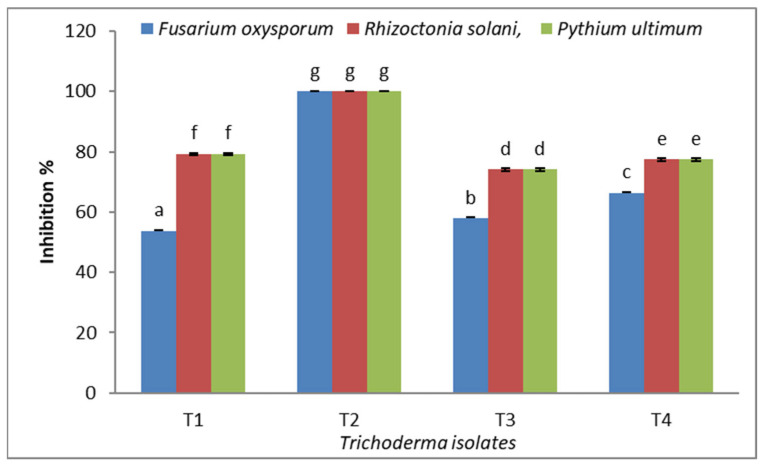
Inhibition percentage of *F. oxysporum*, *R. solani*, and *P. ultimum* by different types of *Trichoderma* spp. Bars represent standard error of the means. Different letters indicate significant value using Duncan’s multiple range test procedure at a significance level of 0.05.

The efficacy of regional *Trichoderma* isolates in PDA medium for preventing the mycelia development of *F. oxysporum* was evaluated. The findings demonstrated that each isolated species of *Trichoderma* considerably and to various degrees hindered the radial development of *F. oxysporum* (Table 1) (Figure 1). The *Trichoderma* isolates possibly prevented the mycelia growth of *F. oxysporum* by between 53.64% and 100%. Maximum mycelia growth inhibition of *F. oxysporum* was exhibited by isolate T2 (100%), followed by T4 (66.28%), while the lowest inhibition (53.64%) was caused by isolate T1. According to the findings, every isolated *Trichoderma* isolate considerably and to various degrees restricted the radial growth of *R. solani*, with *Trichoderma* isolate T2 exhibiting full overgrowth, and the experiment targeted with T3 showed the least inhibition (74.08%). *P. ultimum* mycelia development was successfully restrained by *Trichoderma* isolates; the isolate T2 displayed full overgrowth on *P. ultimum.* The pathogen tested had the lowest percentage of isolate T3 (68.52%) inhibiting mycelia development. *Trichoderma* is a well-known plant pathogen antagonist and a highly efficient biological control agent for a number of soil-borne fungal plant pathogens [20,21]. Particularly in the case of *F. oxysporum* infection, *Trichoderma* spp. can operate as a biological inhibitor of phytopathogenic fungus in several plants [20]. Rojo et al. [21] mentioned that *T. harzianum*, *T. koningii*, and *T. viride* stopped the spread of *Fusarium* spp. and *R. solani* infection in bean seedlings. Also, in soils where these diseases naturally occur, treating sunflower seeds with microorganisms that are harmful to *Rhizoctonia solani*, *Fusarium* spp., and *Macrophomina phaseolina* may prevent their proliferation [22]. Due to competition for scarce resources, which is the most frequent cause of microorganism death, fungal phytopathogens are biologically controlled. According to Howell [23], competition is effective when the pathogen conidia demands further nutrients for germination and germ-tube elongation.

#### 2.1.2. Analysis of the Enzymatic Crude Extract and Antibiotics in Fungal Filtrate of the Most Active *Trichoderma* Isolate

The enzymatic crude extract of isolate T2 was obtained by filtration of the fungal culture by using a sterilized bacterial filter (0.45 µm) under sterilized conditions. The analysis of fungal filtrate took place by enzyme assay in culture filtrate. The results in Table 2 show that T2 produced extracellular enzymes of polygalacturonase (7.56), xylanase (7.30), protease (5.65), β-glucosidase (3.98), cellulase (3.41), B, 1-3-exoglucanse (3.22), and chitinase (1.93 µg/mL). The extracellular enzyme activities produced by three *Trichoderma harzianum* strains in culture filtrates were chitinase (2,60, 3,66, and 4.75 µmol GlcNAc h^−1^ (mg protein)^−1^) and glucanase (67, 24, and 85 µmol glucanase h^−1^ (mg protein^)−1^) after 48 h of fungal cultivations [12]. Several studies have shown that *Trichoderma* spp. cell wall fragments generated by extracellular enzymes during mycoparasitic response can suppress various plant diseases, including *Fusarium* spp. Chitinases and glucanases have a critical role in the mycoparasitism process, as they act as proteolytic enzymes, catalyzing the hydrolysis of peptide bonds in proteins [24]. *Trichoderma* species produce and secrete β-1,3-glucanase, NAGAse, chitinase, acid phosphatase, acid proteases, and alginate lyase, which are effective antagonists against the pathogens [25]. *Trichoderma* β-1,3-glucanases are vital for the enzymatic degradation of the cell walls of phytopathogenic fungi during mycoparasitic attraction [26]. The production of hydrolytic enzymes by *Trichoderma* has been shown to be influenced according to culture conditions and the host [27]. According to [28,29], *Trichoderma* species either indirectly or directly suppress fungal phytopathogens through biological means via competition for resources and space, altering environmental factors, or encouraging the development of plant defenses and antibiosis or directly through processes like mycoparasitism. Our findings demonstrate that extracellular enzymes are released from the *Trichoderma* isolates under examination, and these findings are consistent with those of Chen et al. [30] and Mukherjee et al. [31].

#### 2.1.3. Effect of *Trichoderma* spp. (T2) on Tested Phytopathogenic Fungi under Scanning Electron Microscopy

Mycelial samples from the communication area of the dual culture of *F. oxysporum* plus *Trichoderma* sp. (T2) were observed by scanning electron microscope (Figure S2). Hyphae of *Trichoderma* spp. (T2) frequently grow parallel to the hyphae of the host *F. oxysporum* and stick onto its surface, followed by hasty and excessive coiling (Figure S2) and establishment of appressoria-like structures on the host surface (Figure S2). Finally, lysis of host cell walls was also observed (Figure S2), showing the mycoparasitic nature of *Trichoderma* sp. (T2) on *F. oxysporum.* The *Trichoderma* isolates showed mycoparasitic activity, which was determined using the capacity for *Trichoderma* overgrowth on the mycelia development of *Fusarium oxysporum* in the culture, which could indicate that it can actively parasitize the pathogen.

#### 2.1.4. Screening of Plant Extracts as Antifungal Activities against Tested Fungi

**Figure 2:**
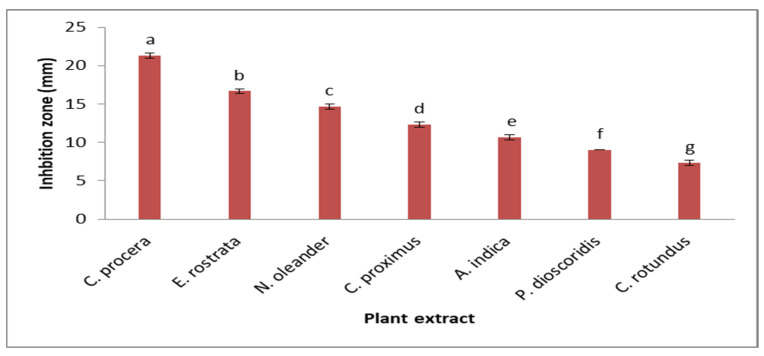
Screening of plant extracts for antifungal activities against *F. oxysporum.* Bars represent standard error of the means. Different letters indicate significant value using Duncan’s multiple range test procedure at a significance level of 0.05.

The antifungal effects of seven plant extracts against *F. oxysporum* were examined (Figure 2). The order of antifungal activities was as follows: *Calotropis procera*, *Eucalyptus rostrata*, *Nerium oleander*, *Cymbopogon proximus*, *Azadicachta indica*, *Pluchea dioscoridis*, and *Cyperus rotundus*, with 21.33, 16.67, 14.67, 12.33, 10.67, 8.67, and 7.33 mm, respectively. The results showed that *C. procera* (21.33 mm) was the most effective plant extract against *F. oxysporum*, and the least effective plant extract was *C. rotundus* (7.33 mm). The strongest and most effective growth inhibitor of fungus among all plant extracts for all tested phytopathogenic species was a plant extract called *Calotropis procera* [32]. Secondary plant metabolites that may prevent pathogen formation and proliferation include polyphenols, alkaloids, flavonoids, and terpenoids, which may explain why plant extracts have antifungal properties [33]. A fungistasis was seen as a result of the *Calotropis procera* plant extract’s impact on the tested fungal development, which prevented the tested fungal mycelia and linear growth. The earlier findings were consistent with those of [34,35]. Numerous secondary metabolites produced by plants have a biocide effect on postharvest infections [36]. These substances have a connection to the plant’s immune system and can be effective fungal inhibitors [37]. The antimicrobial properties of natural plant extracts have been demonstrated in numerous investigations, largely because of their abundance in various phenolic component classes [38]. The thiol group at the plant extract active site is where polyphenols have shown antifungal properties [21].

#### 2.1.5. Effect of Different Concentrations of *Calotropis procera* against Tested Fungi

**Figure 3:**
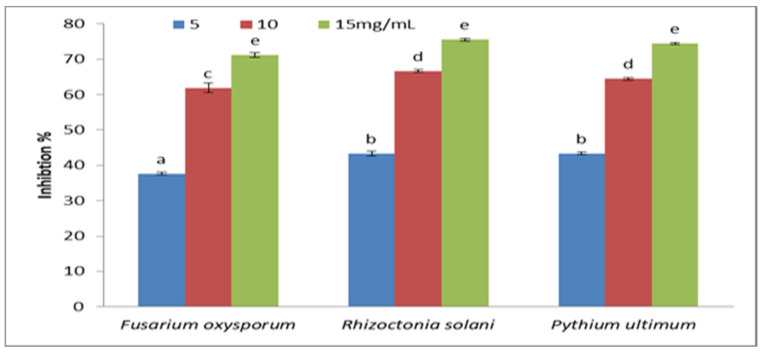
Inhibition percentage of different concentrations of *Calotropis procera* extracts on the growth of *F. oxysporum*, *R. solani*, and *P. ultimum* in vitro. Bars represent standard error of the means. Different letters indicate significant value using Duncan’s multiple range test procedure at a significance level of 0.05.

**Figure 4:**
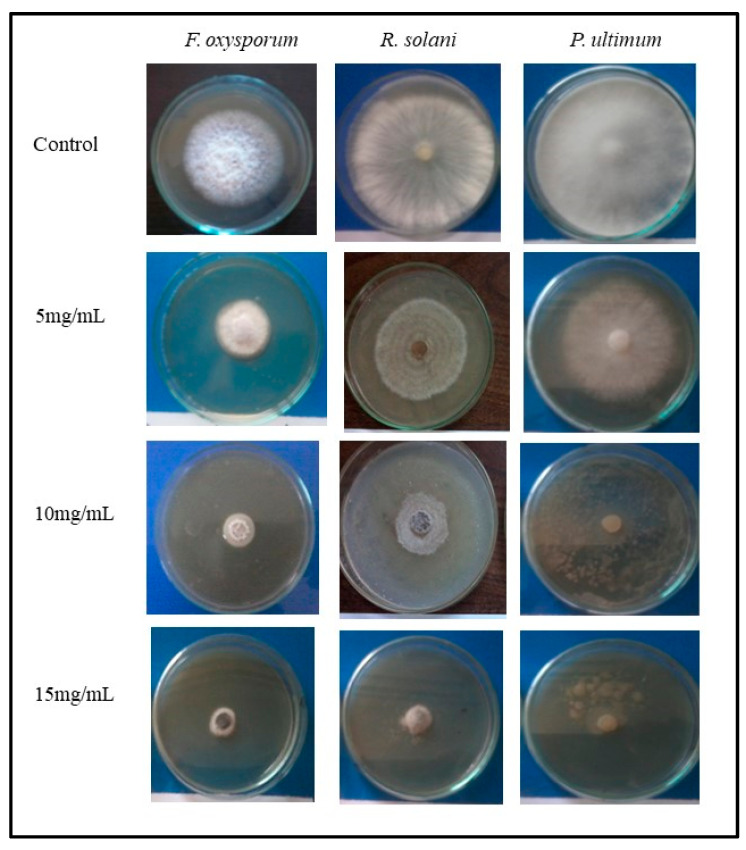
Effect of different concentrations of *C. procera* extracts on the mycelia growth of tested fungi in vitro.

The mean values of growth (colony diameter in mm) of the tested fungi, i.e., *F. oxysporum*, *R. solani*, and *P. ultimum*, as grown on the optimal agar medium containing different concentrations of plant extract of *C. procera* (5, 10, and 15 mg), are represented in Figure 3 and Figure 4. The growth from all treatments was measurable by the end of the 6th day after inoculation. The results revealed that the fungal growth was inhibited by increasing the concentration of the plant extract. Low inhibition percentages were obtained with low concentrations of plant extract (5 mg) that affected each fungi (*F. oxysporum*, 37.64%; *R. solani*, 43.33%; *P. ultimum*, 43.33%). Meanwhile, the inhibition percentage of tested fungi increased by increasing the plant extracts’ concentrations. At 15 mg/mL, the inhibition percentage was 71.13%, 75.55%, and 74.44% for *F. oxysporum*, *R. solani*, and *P. ultimum*, respectively. There were no significant effects of plant extracts on either *R. solani* or *P. ultimum*.

### 2.2. Phytochemical Analysis of Most Active Plant Extract

**Figure 5:**
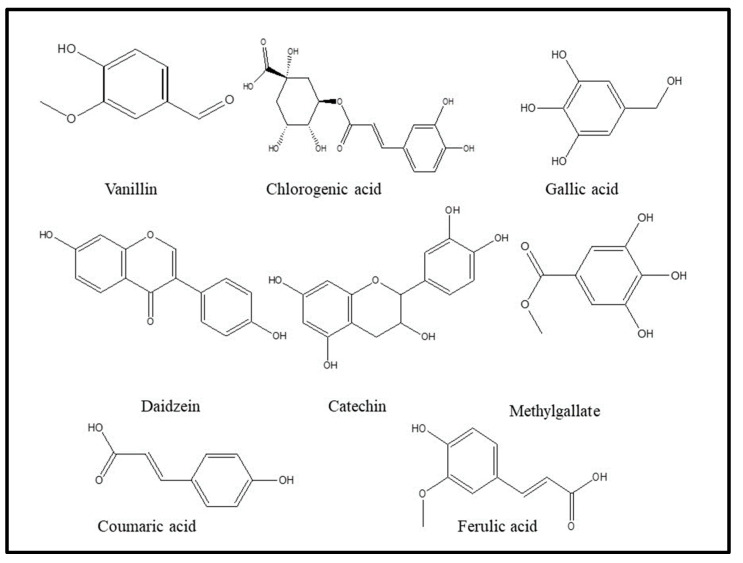
Chemical structures of some phenolic compounds of *Calotropis procera* methanol extract.

Table 3 and Figure 5 show a phytochemical analysis of the methanol extract of *C. procera* obtained by HPLC_UV. The results demonstrate that 18 compounds were found, three of which are flavonoid, plus fifteen phenolic compounds. Flavonoid compounds such as rutin, naringenin, and hesperetin were found at percentages of 1.29, 1.56, and 0.85, respectively, representing only a minimal presence. The major compounds were phenolic, including vanillin (46.79%), chlorogenic acid (30.24%), gallic acid (8.06%), and daidzein (3.45%). All phenolic compounds have antifungal activities, as shown in Table 3.

### 2.3. In Vivo Experiments

#### 2.3.1. Effect of *Trichoderma* spp. filtrate and Methanol Extract of *C. procera* Each or in Combination on Cantaloupe Plants Infected with *F. oxysporum*

##### Disease Incidence

**Figure 6:**
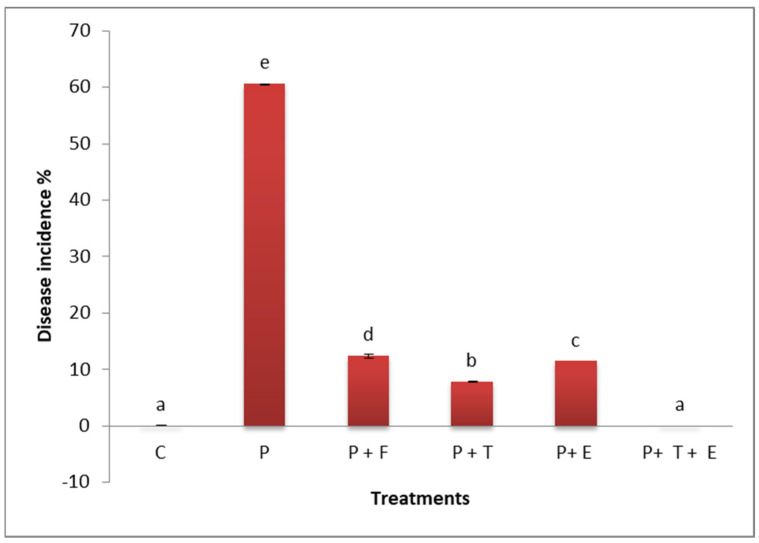
Effect of *Trichoderma* spp. and extract of *C. procera* each and in consortium on disease incidence of cantaloupe plants infected with *F. oxysporum.* P = the pathogen; C = the control (*F. oxysporum*). P + F (pathogen + fungicide), P + T (pathogen + treatment of *Trichoderma* sp.), P + E (pathogen + extract of *C. procera*), and P + T + E (pathogen + *Trichoderma* sp. + extract of *C. procera*). Different letters indicate significant value using Duncan’s multiple range test procedure at a significance level of 0.05.

The data in Figure 6 show that, in comparison to the control plants, *F. oxysporum*-infected cantaloupe plants had a disease incidence (DI) that was extremely high (60.33%). The disease incidence (DI) was reduced when diseased cantaloupe plants were treated with *Trichoderma* spp., *C. procera* extract, *Trichoderma* spp., or *C. procera* extract consortium or the fungicide Rhizolex. The *Trichoderma* spp. and *C. procera* extract consortium was the most successful treatment in lowering the prevalence of wilting disease in cantaloupe plants (0.00%).

Cultural filtrates of the antagonistic fungal strains *Trichoderma harzianum* and *Trichoderma viride* showed antifungal potency against different pathogenic fungal strains [56]. The culture filtrate of *Trichoderma longibrachiatum* SFC100166 could be a valuable source for the enhancement of natural agents to control late blight caused by *Phytophthora infestans* [57].

Many soil-borne fungi, including the *Fusarium* species, *S. sclerotium*, *R. solani*, *S. rolfsii*, *Pythium* species, and *R. solani*, can be controlled by using *Trichoderma* species on vegetables, industrial crops, and fruit [58].

#### 2.3.2. Growth Parameters

**Figure 7:**
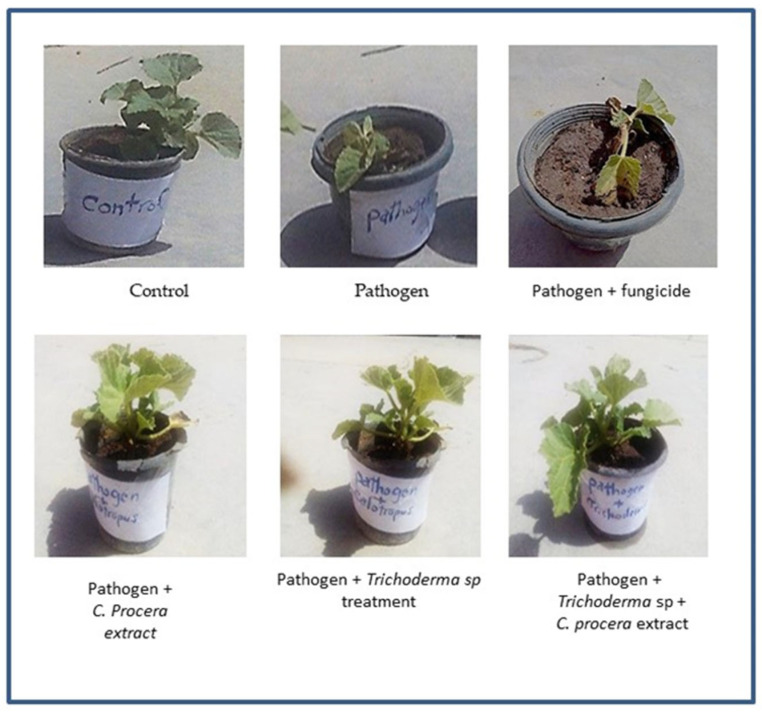
Effect of *Trichoderma* spp. and extract of *C. procera* based on factors for growth; cantaloupe plant diseased with *F. oxysporum*.

The results in Table 4 and Figure 7 demonstrate that in cantaloupe plants that were infected with *F. oxysporum*, all growth indicators were drastically lowered, including shoot fresh weight (4.57 gm), shoot dry weight (2.47 gm), shoot length (14.27 cm), number of leaves (5.33), root length (10.33 cm), root dry weight (0.63 gm), and root fresh weight (1.58 gm), when in comparison to the control plants. The application of *C. procera* extract along with *Trichoderma* spp. considerably improved the growth responses of cantaloupe plants (root length was 23.67 cm, fresh weight was 7.54 gm, dry weight was 3.45 g, shoot length was 28.27 cm, fresh weight was 15.60 g, dry weight was 8.57 gm, and there were 11.00 leaves).

Cantaloupe plants cultivated in soil treated with *Trichoderma* spp. had greater plant height and fresh weight than cantaloupe plants inoculated with *F. oxysporum*, according to the effect of antagonism on their growth under pot conditions. In the current investigation, plants treated with *Trichoderma* spp. also grew taller. *Trichoderma gamsii* application produced similar effects on the growth of cereal and crops of legumes [59]. According to Abou-Zeid [60], inoculating tomato plants with antagonists can decrease the occurrence of several diseases. The best effect in suppressing cantaloupe root rot was shown by the growth of plants treated with *Trichoderma* spp., which was greater than the growth of those under the pathogen alone. In a pot culture assay, it was discovered that adding *Trichoderma* spp. To the soil improved the root and shoot length. A diffusible growth-regulating agent was also used to stimulate increased growth in *Trichoderma* spp., and *T. harzianum* completely prevented *R. solani* infection.

#### 2.3.3. Root Surface Area

**Figure 8:**
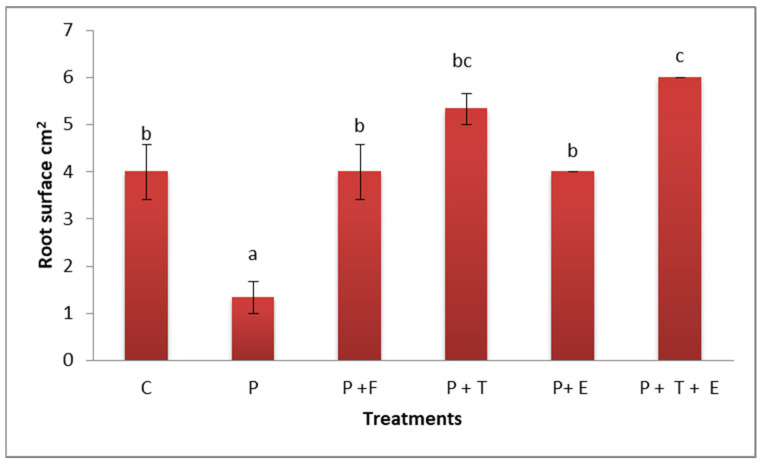
Effect of *Trichoderma* sp. and extract of *C. procera* on root surface area of cantaloupe plants infected with *F. oxysporum*. P = the pathogen; C = the control (*F. oxysporum*). P + F (pathogen + fungicide), P + T (pathogen + treatment of *Trichoderma* sp.), P + E (pathogen + extract of *C. procera*), and P + T + E (pathogen + *Trichoderma* sp. + extract of *C. procera*). Different letters indicate significant value using Duncan’s multiple range test procedure at a significance level of 0.05.

The information reported in Figure 8 illustrates the cantaloupe plant’s root surface area. According to the findings, plants with *F. oxysporum* infection alone had an extremely small root surface area of 1.0 cm^2^ compared to control plants (4 cm^2^). However, the diseased plants treated with *Trichoderma* spp. or *C. procera* extract had greater root surface areas (5 and 4 cm^2^, respectively). However, combining *Trichoderma* spp. and *C. procera* extract led to a very large root surface area (6 cm^2^). The results show significant change between plants infected with the pathogen fungus and other treatments.

#### 2.3.4. Pearson Correlation Analysis

The correlation coefficients (R^2^) and relative *p*-values among the morphological parameters of treated plants show a high correlation among all morphological parameters that were measured, including root length, root fresh weight, root dry weight, shoot fresh weight, shoot dry weight, leaf numbers, and root surface area (Table 5).

#### 2.3.5. Total Phenol Content

**Figure 9:**
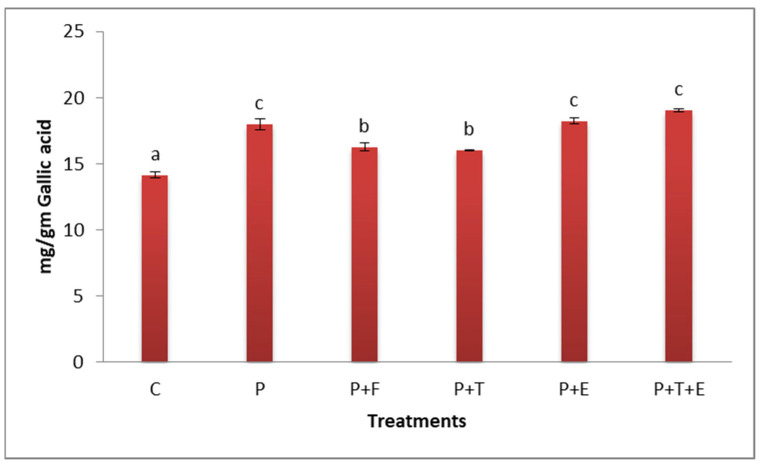
Effect of *Trichoderma* spp. and extract of *C. procera* both alone and in consortium on total phenol contents of cantaloupe plants infected with *F. oxysporum.* P = the pathogen; C = the control (*F. oxysporum*). P + F (pathogen + fungicide), P + T (pathogen + treatment of *Trichoderma* sp.), P + E (pathogen + extract of *C. procera*), and P + T + E (pathogen + *Trichoderma* sp. + extract of *C. procera*). Different letters indicate significant value using Duncan’s multiple range test procedure at a significance level of 0.05.

Total phenol contents were examined in cantaloupe plants that were infected and treated with *Trichoderma* spp., *C. procera* extract, or the fungicide Rizolex-MZ. Despite the elevated total phenol content in *F. oxysporum*-infected cantaloupe plants, treatment with *Trichoderma* spp., *Calotropis procera* extract, or the fungicide Rizolex-MZ resulted in a further increase in the phenol content. The treatment of *Trichoderma* spp. with *C. procera* extract resulted in the greatest increase in the phenol content of infected cantaloupe plants (19.27 µg·g^−1^ gallic acid) (Figure 9). There were no significant effects among plants infected with the pathogen and the plants infected with the pathogen and treated with *C. procera* both alone and with *Trichoderma* spp.

It was hypothesized that *Trichoderma* inoculation raised the lytic enzyme’s activity, which in turn increased the quantity of phenols. This may have increased physical barrier strength or created a chemical barrier that was resistant in comparison to the hydrolytic enzymes created by the infection, leading to resistance against *F. oxysporum* [61].

## 3. Materials and Methods

### 3.1. Isolation, Purification, and Identification of F. oxysporum, P. ultimum, and R. solani

Cantaloupe plants with varying degrees of root and wilt were collected in Egypt from many locations, including Sadat City, Nubaria, El Khatatba, and Alexandria (K. 76 Egypt Road). The infected roots were cut into small pieces, properly cleaned with running tap water, and then immersed in sodium hypochlorite (0.5% chlorine) for one minute. The surface was cleaned using D. water and dried between two sterile filter sheets [62]. The roots were cut into small, 2–3 mm pieces, and four pieces were placed on a 2.5% potato dextrose agar (PDA) medium amended with 10 mg L^−1^ rifampicin and 200 mg L^−1^ ampicillin. The plates were maintained at ambient temperature for 7 to 10 days and then at 28 °C for 7 days. Pure cultures were kept for future use at 5 °C, and the separated fungi were identified using cultural and morphological characteristics as well as microscopically described [63]. The isolates were identified as *Fusarium oxysporum* 9704 AUMC, *Rhizoctonia solani* 6590 AUMC, and *Pythium ultimum* 4413AUMC, according to Booth [64]. The identification was performed by the Mycology Center, Assiut University (AUMC).

### 3.2. Isolation and Identification of Trichoderma spp.

There are many ways to isolate *Trichoderma* spp.; however, serial dilution of samples is one of the most frequently described ways in the literature [65,66]. This procedure is easy, inexpensive, and suitable for handling big samples. Soil samples were collected around healthy roots of cantaloupe plants from fields across Egypt (Table 6), including Sadat City, Nubaria, El Khatatba, and K. 76 Egypt Road, Alexandria. This was followed by air drying and grinding soil samples into powder. The sample stock solution was produced by mixing 10 g of powdered soil sample with 90 mL of distilled water. The samples were then serially diluted, as 10^−1^, 10^−2^, 10^−3^, 10^−4^, 10^−5^, and 0.5 mL of each prepared dilution was equally dispersed and kept for seven days at 28 °C on a PDB medium in a Petri plate. The isolated fungi were sub-cultured in *Trichoderma*-selective (TSM) medium and identified based on physiological, morphological, and cultural features [67]. *Trichoderma* spp. isolates were identified based on conidiophores, phialides, mycelium structure, growth, and other colonial characteristics as well as conidia [68]. The identity of the chosen *Trichoderma* spp. isolate was confirmed by the Mycology Center at Assiut University in Egypt.

### 3.3. Collection of Wild Plants, Identification, and Extractions

Seven wild plants, namely *Eucalyptus rostrata* Schlecht, *Pluchea dioscoridis* L, *Nerium oleander* L., *Cymbopogon proximus* (hochst) Staps, *Azadicachta indica* Adrjuss, *Calotropis procera* (Aiton), and *Cyperus rotundus* L., were collected from fields, canal sides, and desert region of Sadat City in different regions of the El-Menofya governorate [69]. Ten grams of plant leaf powder was steeped for one week in 100 mL of 70% methanol at room temperature in an orbital shaker (120 rpm). The mixtures were then centrifuged at 5000 rpm for 5 min. Using a rotary evaporator, the supernatants were evaporated. The plant extract was diluted in 1% dimethyl sulfoxide (DMSO) to obtain a final concentration of 15 mg/mL [70].

### 3.4. Disease Control (In Vitro Experiments)

#### 3.4.1. The Inhibitory Activity of *Trichoderma* spp. Isolates versus Tested Phytopathogenic Fungi (Dual-Culture Experiments)

By using a dual-culture method, the antagonistic action of *Trichoderma* spp. isolates versus the tested fungi was assessed [71]. A pathogen disk (6 mm in diameter) was located on one side of the Petri dish. On the parallel side, an antagonist disk was located an even distance apart. PDA plates containing only the pathogen’s mycelium’s disks were used as controls. Each experiment was performed three times. The phytopathogens’ radial growth in the control and treatment plates was assessed after six days of incubation at 28 °C, and the percentage of inhibition of radial mycelial growth (PIRG) was determined using the following formula:

PIRG % = (R1 − R2)/R1 × 100

where R1 = radial growth of the phytopathogen in the control plate; R2 = radial growth of the phytopathogen when an antagonist is present [72].

#### 3.4.2. Enzymes Assays and Antibiotics of Selected *Trichoderma* spp. Isolate in Culture Filtrate

##### Enzymes Assays

Enzyme assay experiments were performed in the Central Labs Unit of Research National Center, Egypt. Chitinase activity was measured according to the methods of Boller [73] and Reissig [74]. Cellulase activity and polygalacturonase (PG) activity were measured in relation to the method of Collmer [75] and Miller et al. [76]. “β-1,3-glucanase” activity was determined by the Malik and Singh [77] methods. Protease activity was assessed according to methods of Lee and Takahashi [78]. Xylanase activity was evaluated related to the methods of Bailey et al. [79], and β-glucosidase activity was measured in relation to the Berghem and Petterson [80] methods. (The methods are presented in detail in the Supplementary Materials).

### 3.5. Sample Preparation for Scanning Electron Microscopy (SEM)

A scanning electron microscope (SEM) was used to examine the hyphal interaction between *F. oxysporum* and *T. atroviride*. A mycelial disc (5 mm) was inserted into the PDA plate, taken from both colonies of the leading edge of *F. oxysporum* and *T. atroviride*, both of which grow toward each other and intermix their hyphae, in order to locate the hyphae interaction areas. Agar blocks measuring 1 cm thick were removed from plate culture, and the interaction areas were marked for SEM processing. Leica’s tissue processor model Lynexel was used for sample preparation. The mycelial samples from the contact region were first fixed with osmium oxide before being dehydrated with ethyl alcohol serial dilution followed by acetone. The treated samples were then processed again after being coated with gold using a sputter coater (EMS 550) and dried using a critical point drier (EMS 850). Then, by using an SEM (JEOL100CX-ASID-4D), the mycoparasitism and hyphae contacts were examined at the EM Unit in the Mycology Center at Al-Azhar University in Egypt, the microscope was run at 30 kV [81].

### 3.6. Antifungal Screening of Selected Plant Extracts

The agar well diffusion method was used to screen the tested seven plant extracts for their antifungal activities, as demonstrated by Daoud [82]. A sterile Petri dish was pipetted with one milliliter of 10^6^ conidia·mL^−1^ 6–7-day-old *F. oxysporum* culture in the center. The medium of PDA for fungi was then poured into the Petri dish containing the inoculum and mixed well. Using a sterile cork borer (6 mm in diameter), wells were formed into agar plates containing inoculums after hardening. Then, 100 μL of each extract were applied to the appropriate wells at a concentration of 15 mg/mL. The plates were chilled for 30 min to ensure that the extracts were mixed effectively with the agar. After that, the plates were incubated for 48 h at 28 °C. After measuring the diameter of the inhibition zone, which included the well diameter, the following the incubation period allowed for the detection of antifungal activity. DMSO at a concentration of 10% was used as a negative control.

### 3.7. In Vitro Evaluation of the Effect Different Concentrations of C. procera against Tested Fungi

The experiment evaluated the effects of different concentrations of the plant extract of *C. procera* (5, 10, and 15 mg/mL) on mycelial growth of the tested fungi, i.e., *F. oxysporum*, *P. ultimum*, and *R. solani*. The tested phytopathogenic fungi were cultivated for five days at 25 °C on PDA before being used for the radial growth tests. In the center of a PDA plate, 20 μL of each concentration of *C. procera* extract was then added. After the treatments were absorbed into the agar, a 5 mm diameter plug from the PDA fungal cultures was inoculated on the center of the plate. Every assay was performed three times. Then, the cultures were incubated at 28 °C for 5 days. The inhibitory activity or radial growth (IR) was calculated according to the following formula [83]:

% IR = Dc − Dt/Dc × 100

where IR = inhibiting mycelial development percentage; Dc = average fungal mycelial growth diameter of the DMSO (20 μL) at a concentration of 10%, which was employed as a negative control [81]; Dt = average fungal mycelial growth diameter after extract treatment.

### 3.8. Phytochemical Analysis of Most Active Plant Extract by HPLC UV–Vis Detectors

The phytochemical compositions of the extract from leaves of *Calotropis procera* were determined using the *HPLC UV–Vis Detectors* to find phenolic, flavonoids, and other active compounds, and analysis was performed with an Agilent 1260 series instrument. The analysis was conducted in the Central Labs Unit of the Research National Center, Egypt. Using a Zorbax Eclipse Plus C8 column (4.6 mm × 250 mm i.d., 5 μm), the separation was performed. At a flow rate of 0.9 mL/min, the mobile phase was composed of water (A) and 0.05% trifluoroacetic acid in acetonitrile (B). The following was the sequential linear gradient programming for the mobile phase: 0 min (82% A), 0–1 min (82% A), 1–11 min (75% A), 11–18 min (60% A), 18–22 min (82% A), and 22–24 min (82% A). At 280 nm, the multi-wavelength detector was observed. For every sample solution, there was one injection volume of five microliters. At 40 °C, the column temperature was kept constant.

### 3.9. Disease Control (In Vivo Experiments)

#### Effects of *Trichoderma* spp. filtrate and *C. procera* plant extract on cantaloupe plants infected by *F. oxysporum*

To obtain the inoculum of *Fusarium oxysporum*, five discs of a seven-day-old *F. oxysporum* culture grown on PDA medium were added to 500 mL bottles of sand-cornmeal medium (SCM), and the mixture was incubated at 28 °C for 14 days [84]. Autoclave sterilization was used to prepare the 1:2 sand-clay soil used in this experiment. Then, 1 gm/kg soil *F. oxysporum* inoculum was added to pots (12 cm in diameter by 20 cm in height) containing 2.5 kg of sterilized soil; they were mixed well in upper surface and then watered as necessary for 7 days [85]. The healthy root systems of transplanted cantaloupes (30 days old) were soaked in treatments of each of the following: 15 mg/m of *Calotropis procera* extract, *Trichoderma* spp., and its combinations for 30 to 60 min; the plants were then cultivated in the infected soil. The treatments (15 plants in each treatment, one plant in each pot) were according to the following: P = pathogen (*F. oxysporum*), C = control (un-infected soil), P + T = (pathogen + soaking in 10% filtrate of *Trichoderma* spp. (8 days)), P+ F = pathogen + soaking in the fungicide Rizolex-T, and P + T + E (pathogen + soaking in 10% filtrate of *Trichoderma* spp. (8 days) + 15 mg/mL extract of *Calotropis procera*). For the control roots, water was used. In this experiment, the Ministry of Agriculture’s recommended dosage for the fungicide Rizolex-T was 3 gm/kg soil [86]. The data from this experiment were collected after 30 days from the transfer.

### 3.10. Measurements of the Growth Parameters of the Cantaloupe Plants

Fifteen plants were taken from each treatment and cleaned under running water to remove dirt. The length of the root (cm), the length of the shoot (cm), the number of leaves, the fresh and dry weights of the root and shoot (gm), and root surface area (cm^2^) were measured according to Tagliavini [87]. The incidence rate percentages were investigated according to the following equations:

$$\mathrm{Disease}\;\mathrm{incidence}\;(\mathrm{DI})\;\%=\frac{Number\;of\;diseased\;plants}{Total\;number\;of\;plants}\times 100$$

### 3.11. Total Phenol Estamations

Total phenol estimation (mg/gm gallic acid) was carried out using the Folin–Ciocalteau reagent, whereby 0.5 mL of extract was added to 2.5 mL of 10-fold diluted Folin–Ciocalteu reagent and then 2 mL of 7.5% Na_2_CO_3_ solution. The mixture was kept in the dark for 30 min at ambient temperature, and the absorbance was read by spectrophotometry (Helios UV–Vis Scanning Spectrophotometers, Caerphilly, England) 760 nm [85].

### 3.12. Statistical Analysis

The mean of determinations performed in triplicate is the total of all values according to the latest release of SPSS 16. The study employed one-way analysis of variance (ANOVA) to statistically analyze the data. The least significant difference (LSD) is determined at the *p* ≤ 0.05 level.

## 4. Conclusions

As a result, our research was meant to replace the unfavorable and risky usage of pesticides with an alternative non-chemical, ecologically friendly, efficient, biological control action against *Rhizoctonia solani*, *Fusarium oxysporum*, and *Pythium ultimum*, which infect cantaloupe plants under both in vitro and in vivo tests. The results demonstrate that *Trichoderma* isolates have antifungal activities against *F. oxysporum*, *R. solani*, and *P. ultimum* in vitro. *Trichoderma* spp. culture filtrates contain many vital enzymes and antibiotics that are responsible for suppressing the pathogenic fungi. The methanol extract of *Calotropis procera* showed more antifungal activities against *F. oxysporum* among the tested plants. Vanillin, chlorogenic acid, and gallic acid were the most common phenolic compounds found in the methanol extract of *C. procera*. *Trichoderma* spp. culture filtrates in consortium with a methanol extract of *C. procera* reduced the prevalence of wilting disease caused by *F. oxysporum*, enhancing growth parameters such as root length, root fresh, dry weight, shoot length, shoot fresh dry weight, and number of leaves.

## Figures and Tables

**Table 1:** Effect of different *Trichoderma* isolates on the mycelial growth of *F. oxysporum*, *R. solani*, and *P. ultimum* in vitro, with mean radial growth (mm).

Isolates	*F. oxysporum*	*R. solani*	*P. ultimum*
Control	87.00 ± 0.57 a	90.00 ± 0.0 a	90.00 ± 0.0 a
T1	40.33 ± 0.33 b	18.67 ± 0.33 d	22.67 ± 0.33 d
T2	0.0 ± 0.0 e	0.0 ± 0.0 e	0.0 ± 0.0 e
T3	36.67 ± 0.33 c	23.33 ± 0.33 b	28.33 ± 0.33 b
T4	29.33± 0.33 d	20.33± 0.33 c	25.67± 0.0 c
LSD	0.001	0.001	0.001

Each value is the three-replicate average. Means with the same alphabetical letter in the column within a comparable set of means do not significantly differ from one another when using Duncan’s multiple range test procedure at a significance level of 0.05.

**Table 2:** Extracellular enzymes secreted by *Trichoderma* isolate (T2).

Enzymes of *Trichoderma* Isolate (T2)	(µmol Enzyme min^−1^·mg^−1^ Protein)	Enzymes	(µmol Enzyme min^−1^·mg^−1^ Protein)
Protease	5.65	Polygalacturonase (PG)	7.56
β-1-3-exoglucanase	3.22	β-glucosidase	3.98
Chitinase	1.93	Xylanase	7.30
Cellulase	3.41	Trichorzins PA (peptaibols) μg/mL	13.0

**Table 3:** HPLC UV–Vis Detectors analysis of methanol extract of *Calotropis procera*.

RT	Compound	Type	Area	Area%	Antifungal Activity Against	Ref.
3.58	Gallic acid	Phenolic	162.19	8.06	*Alternaria solani*	[39]
4.27	Chlorogenic acid	Phenolic	608.01	30.24	*Candida albicans*	[40]
4.61	Catechin	Phenolic	11.24	0.55	*Candida albicans*	[41]
5.69	Methyl gallate	Phenolic	32.62	1.62	*Magnaporthe grisea*, *Botrytis cinerea*, and *Puccinia recondita*	[42]
6.39	Syringic acid	Phenolic	20.84	1.03	*Ganoderma boninense*	[43]
6.70	Pyrocatechol	Phenolic	17.34	0.86	*Bipolaris carbonum*	[44]
6.86	Rutin	Flavonoid	26.09	1.29	*Fusarium solani*	[45]
7.47	Ellagic acid	Phenolic	5.76	0.28	*Candida krusei* and *Candida parapsilosis*	[46]
8.68	Coumaric acid	Phenolic	2.16	0.10	*Botrytis cinerea*	[47]
9.25	Vanillin	Phenolic	940.70	46.79	*Alternaria alternata*	[48]
9.85	Ferulic acid	Phenolic	46.86	2.33	*Fusarium graminearum*	[49]
10.5	Naringenin	Flavonoid	31.56	1.56	*Candida albicans*	[50]
11.5	Rosmarinic acid	Phenolic	2.67	0.13	*Candida albicans*	[51]
15.6	Daidzein	Phenolic	69.51	3.45	*Herpes simplex* and *Candida albicans*	[52]
17.3	Quercetin	Phenolic	2.79	0.13	*Candida albicans*	[51]
19.1	Cinnamic acid	Phenolic	11.14	0.55	*Aspergillus flavus*, *Aspergillus terreus*, and *Aspergillus niger*	[53]
20.5	Kaempferol	Phenolic	1.74	0.08	*Fusarium oxysporium*	[54]
21.0	Hesperetin	Flavonoid	17.21	0.85	*Candida albicans* and *Candida tropicalis*	[55]

**Table 4:** Effect of *Trichoderma* spp. and extract of *C. procera* on growth parameters of cantaloupe plants infected with *F. oxysporum*.

Treatment	Growth Parameters						
	R.L. (cm)	R.F.W. (gm)	R.D.W. (gm)	S.L. (cm)	S.F.W. (gm)	S.D.W. (gm)	Leaf Numbers
C	20.67 ± 0.33 b	5.29 ± 0.22 b	2.59 ± 0.19 b	27.33 ± 0.30 b	14.10 ± 0.25 b	7.12 ± 0.0.20 b	9.67 ± 0.27 b
P	10.33 ± 0.33 d	1.58 ± 0.22 d	0.63 ± 0.19 c	14.27 ± 0.30 e	4.57 ± 0.25 d	2.47 ± 0.20 d	5.33 ± 0.27 e
P + F	16.67 ± 0.33 c	3.58 ± 0.22 c	2.33 ± 0.19 b	24.33 ± 0.30 c	12.33 ± 0.25 c	5.53 ± 0.20 c	7.33 ± 0.27 d
P + T	21.67 ± 0.33 b	4.09 ± 0.22 c	2.46 ± 0.19 b	26.43 ± 0.30 b	13.43 ± 0.25 b	6.69 ± 0.20 b	9.00 ± 0.27 b
P + E	16.33 ± 0.33 c	3.57 ± 0.22 c	2.12 ± 0.19 b	23.23 ± 0.30 d	12.53 ± 0.25 c	5.56 ± 0.20 c	8.67 ± 0.27 c
P + T + E	23.67 ± 0.33 a	7.54 ± 0.22 a	3.45 ± 0.19 a	28.27 ± 0.30 a	15.60 ± 0.25 a	8.57 ± 0.20 a	11.00 ± 0.27 a
LSD	0.001	0.001	0.001	0.001	0.001	0.001	0.00

The data that were recorded are the averages of three replicates. Means with the same alphabetical letter in the column within a comparable set of means do not significantly differ from one another when using Duncan’s multiple range test procedure at a significance level of 0.05. P = the pathogen; C = the control (*F. oxysporum*). P + F (pathogen + fungicide), P + T (pathogen + treatment of *Trichoderma* sp.), P + E (pathogen + extract of *C. procera*), and P + T + E (pathogen + *Trichoderma* sp. + extract of *C. procera*).

**Table 5:** Pearson correlation among the plant growth parameters.

	Root Length	Root Fresh Weight	Root Dry Weight	Shoot Length	Shoot Fresh Weight	Shoot Dry Weight	Leaf Numbers	Root Surface
Root length	1							
Root fresh weight	0.890 **	1						
Root dry weight	0.909 **	0.877 **	1					
Shoot length	0.946 **	0.830 **	0.914 **	1				
Shoot fresh weight	0.925 **	0.847 **	0.924 **	0.984 **	1			
Shoot dry weight	0.968 **	0.929 **	0.927 **	0.952 **	0.954 **	1		
Leaf numbers	0.919 **	0.909 **	0.854 **	0.889 **	0.905 **	0.951 **	1	
Root surface	0.884 **	0.759 **	0.851 **	0.852 **	0.857 **	0.858 **	0.816 **	1

** Correlation is significant at the 0.01 level (2-tailed).

**Table 6:** *Trichoderma* isolates from Cantaloupe.

Isolates	Latitude and Longitude	Location of Host Plant
T1	30°38′41.9″ N 30°06′53.9″ E	Nubaria
T2	30°39′37″ N 30°04′03″ E	Sadat City
T3	30°13′04″ N 30°51′51″ E	El Khatatba
T4	30°42′01″ N 30°02′50″ E	Alex. Cairo Road K. 76.

## Data Availability

Data are contained within the article and Supplementary Materials.

## Supplementary Materials

The following supporting information can be downloaded at: https://www.mdpi.com/article/10.3390/molecules29010139/s1, Figure S1: Effect of *Trichoderma* isolates on *F. oxysporum, R. solani*, and *P. ultimum* mycelial growth after 6 days of incubation; Figure S2: Scanning electron microscopy observations of the mycoparasitic nature of *Trichoderma* sp. (T2) on *F. oxysporum.* (A) Parallel growth of *Trichoderma* hyphae (T) and coleogenesis of (T) surrounding (F) hyphae. (B) Formation of structures resembling appressoria (ab) as a result of *Trichoderma* growth on *Fusarium* hyphae (F) sticking together, “Ab”. (C) The *Fusarium* (F) wall finally lysed; Figure S3: HPLC-MS analysis of methanol extract of *Calotropis procera*.

## Abbreviations

PDA, potato dextrose agar; AUMC, Mycology Center, Assiut University; PDB, potato dextrose broth; TSM, *Trichoderma*-selective medium; DMSO, dimethyl sulfoxide; PIRG, percentage of inhibition of radial mycelial growth; PG, polygalacturonase; SEM, scanning electron microscope; LSD, least significant difference; NAGAse, N-Acetyl-β-D-glucosaminidase; DI, disease incidence.
