# Supplementary material for: Polyphenols-Rich Extract of Calotropis procera Alone and in Combination with Trichoderma Culture Filtrate for Biocontrol of Cantaloupe Wilt and Root Rot Fungi
Source: Molecules. 2023 Dec 26;29(1):139. doi: 10.3390/molecules29010139 (PMC10780250; doi:10.3390/molecules29010139)
Supplement: Supplementary file 1 [file molecules-29-00139-s001.zip › molecules-2736301-supplementary.pdf]

Polyphenols-Rich Extract of *Calotropis procera* Alone and in Combination with Trichoderma Culture Filtrate for Biocontrol of Cantaloupe Wilt and Root Rot Fungi

(Supplementary Materials)

Materials and Methods

Enzymes assays

Chitinase activity

The reaction mixture contained 200 µL of 0.5% chitin in 10 mM sodium acetate buffer (pH 5.2), and 100 µL of culture supernatants (Boller, Mauch, 1988) were incubated for 1 h at 50 °C. The formation of sugar *N*-acetylglucosamine was measured by dimethylamino benzaldehyde (DMAB) method (Reissig et al., 1955). A known aliquot of the reaction mixture (0.5 mL) was added to a test tube, and 0.5 ml of 120 mM potassium borate buffer (pH 8.9) was also added. The tubes were vigorously boiled in a water bath for 3 min and cooled. Then, 3 ml DMAB reagent (5.0 g DMAB dissolved in 500 mL of glacial acetic acid containing 12.5 % *v/v* 10 N HCl, stored at 20 °C as a stock, and diluted prior to use with nine volume of glacial acetic acid) was added to each tube and incubated at 38 °C for 20 min. The tubes were then cooled, and absorbance was measured at 544 nm with the spectrophotometer. Standard N-acetylglucosamine was prepared in borate buffer and measured following the above procedure. The amount of N-acetylglucosamine was calculated and expressed as appropriate.

Cellulase activity

A suitable aliquot (100 µL) of the cultured supernatant was incubated with 400 µL of 100 mM sodium citrate buffer (pH 5.2) containing 1 % CMC (Collmer, et al., 1988). After incubation at 55 °C for 15 min, the glucose released was measured by the dinitrosalicylic acid (DNSA) method (Sadasivam, and Manickam, 1992). A known volume of aliquot was added to a test tube, and a final volume of 1.0 mL was reached with distilled water. To this, 0.5 mL DNSA reagent (1 g DNSA + 200 mg crystalline phenol + 50 mg sodium sulfite in 100 mL of 1% sodium hydroxide) was added and mixed properly. The content was heated in a boiling water bath for 5 min. When the contents of the tubes were still warm, 1.0 mL of 40% sodium potassium tartrate (Rochelle salt) solution was added and cooled. The final volume of 5.0 mL was obtained with distilled water and read at 540 nm using a spectrophotometer. A reagent blank was also performed by addition of 1.0 mL of distilled water in place of the enzyme aliquot and treated in the same way as in the above procedure. A known concentration of standard of glucose was calibrated by following the above procedure and the enzyme activity expressed as appropriate.

Poly galacturonase

The culture supernatants (100 µL) were incubated with 400 µL of 50 mM sodium acetate buffer (pH 5.2) containing 0.25% sodium polypectate (Collmer, et al., 1988). After incubation at 37 °C for 1 h, the galacturonic acid released was measured by the DNSA method (Sadasivam, and Manickam, 1992).

β-1, 3 glucanase activity

The reaction system contained 100 µL of 4% laminarin in 50 mM sodium acetate buffer (pH 5.2) and 100 µL of culture supernatants (Kauffman, et al., 1987). Reactions were carried out at 37 °C for 10 min. After incubation, the glucose released by enzyme b-1,3 glucanase was measured by DNSA method (Sadasivam, & Manickam, 1992).

The specific activity of cellulase, PG, chitinase, and b-1,3 glucanase is expressed as unit.mg^{-1} protein. However, unit activity was defined as the amount of enzyme necessary to produce 1 µM of corresponding reducing sugar per minute per milliliter of culture supernatants. Non-enzymatic controls were also performed using boiled enzymes and were subtracted from the enzymatic values.

Protease activity (EC 3.4.21.4)

The reaction system contained 500 µl enzyme solution and 500 µL of 0.36% casein and 2.0 mL of 100 mM acetate buffer (pH 3.6). The reactions were allowed to proceed for 1 h at 50 °C and were stopped with 3 mL of 5% trichloroacetic acid (Malik, Singh, 1980). The blank was treated as zero time incubation. The reaction mixtures were then centrifuged at 5000 rpm for 10 min to settle the precipitate, and a known volume of supernatants (500 µL) was used for the estimation of released free amino acids following the ninhydrin method (Lee, Takahashi, 1966). The specific activity of protease is expressed as unit.mg^{-1} protein, and one unit of protease activity is defined as the amount of protein necessary to produce 1 µg free amino acids per minute per milliliter of culture supernatant.

Xylanase activity

Xylanase activity was assayed by the method described by Bailey et al. [Bailey, et al. 1992]. Oat spelt xylan (Sigma-Aldrich, St Louis, MO, USA) was used as the substrate. The amount of released sugar was assayed via the dinitrosalicylic acid (DNS) method using glucose or xylose as the standard [Miller et al., 1960,].

β-Glucosidase activities

β-Glucosidase activities were determined using 4-nitrophenyl-β-D-glucopyranoside with para-nitrophenol as the standard [Berghem and Petterson, 1974]. One unit (U) of enzyme activity was defined as the quantity of enzyme that liberated the substrate at the rate of 1 µmol per minute.

Antibiotic

After induction, the medium was separated from mycelia by vacuum filtration, and the peptides were extracted by adding ethyl acetate (1:2 *v/v*, Dinâmica). After phase separation in a separating funnel, the apolar phase was collected, centrifuged, and rotoevaporated. The residue formed was resuspended by washing with water and then with acetonitrile and collected separately. The acetonitrile fraction was lyophilized and used as a peptaibol source.

	F. oxysporum	*R. solani*	*P. ultimum*
**Control**	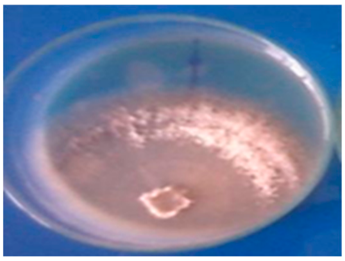	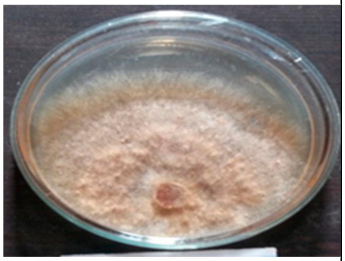	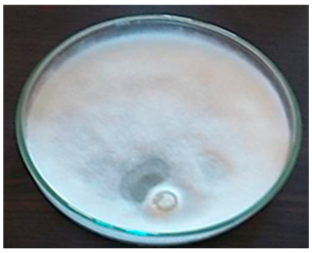

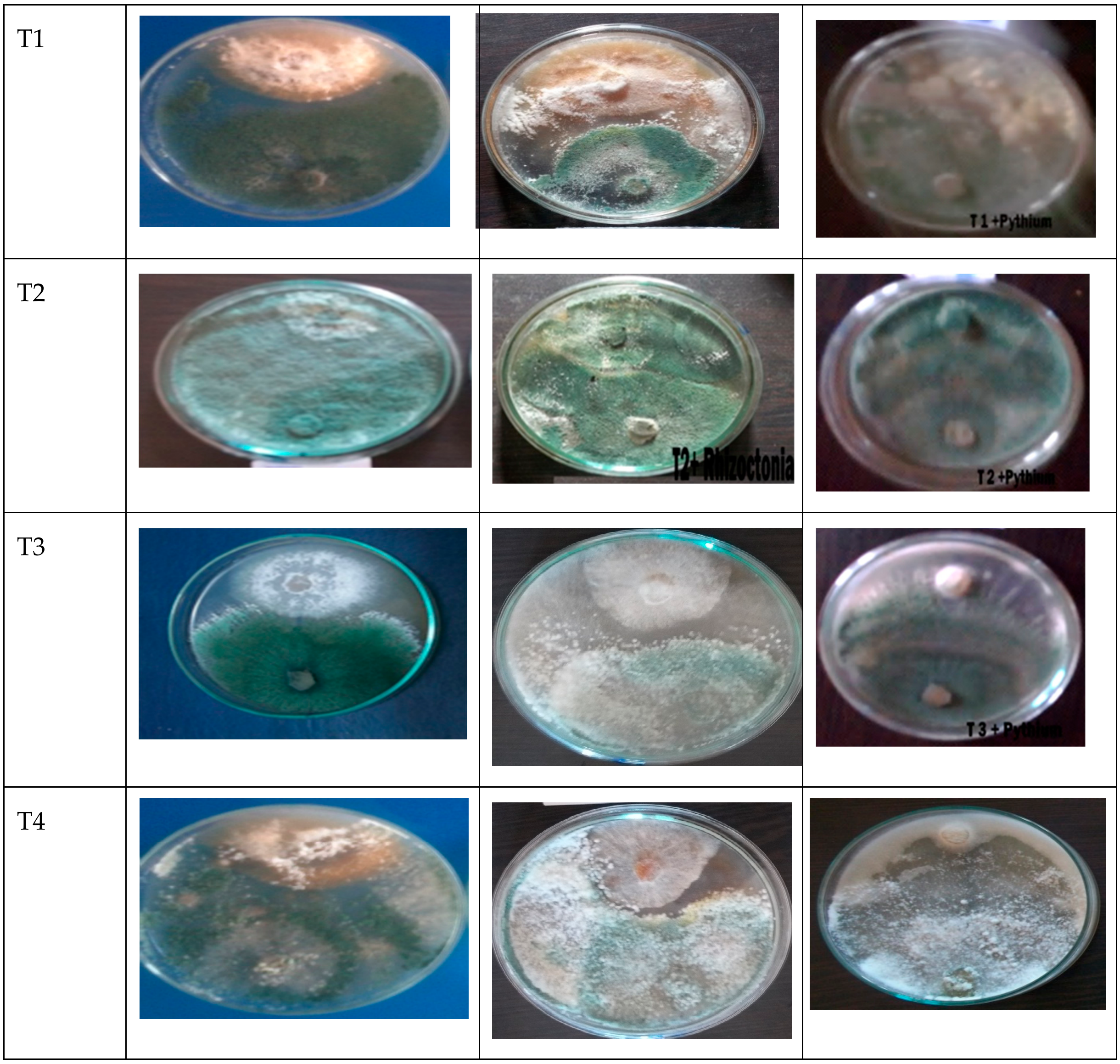

Figure S1. Effect of *Trichoderma* isolates on *F. oxysporum, R. solani*, and *P. ultimum* mycelial growth after 6 days of incubation.

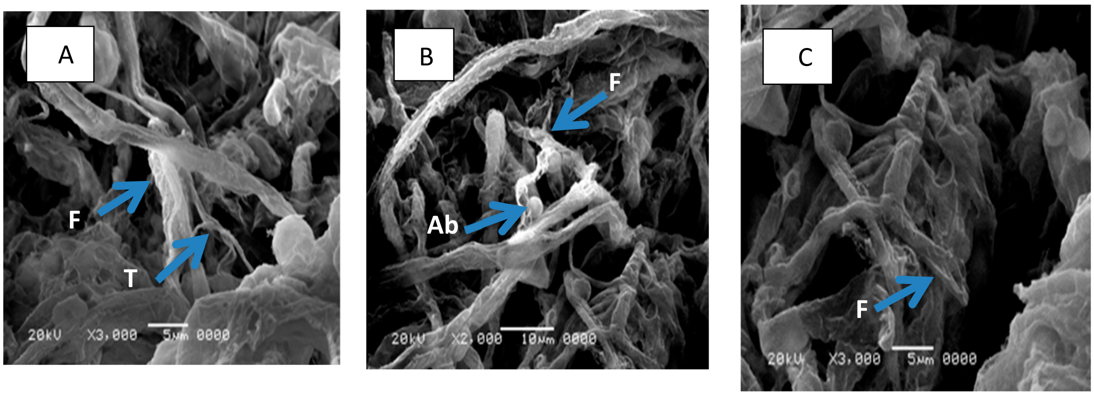

Figure S2. Scanning electron microscopy observations of the mycoparasitic nature of *Trichoderma* sp. (T2) on *F. oxysporum*. (**A**) Parallel growth of *Trichoderma* hyphae (T) and coleogenesis of (T) surrounding (F) hyphae. (**B**) Formation of structures resembling appressoria (ab) as a result of *Trichoderma* growth on *Fusarium* hyphae (F) sticking together, "Ab". (**C**) The *Fusarium* (F) wall finally lysed.

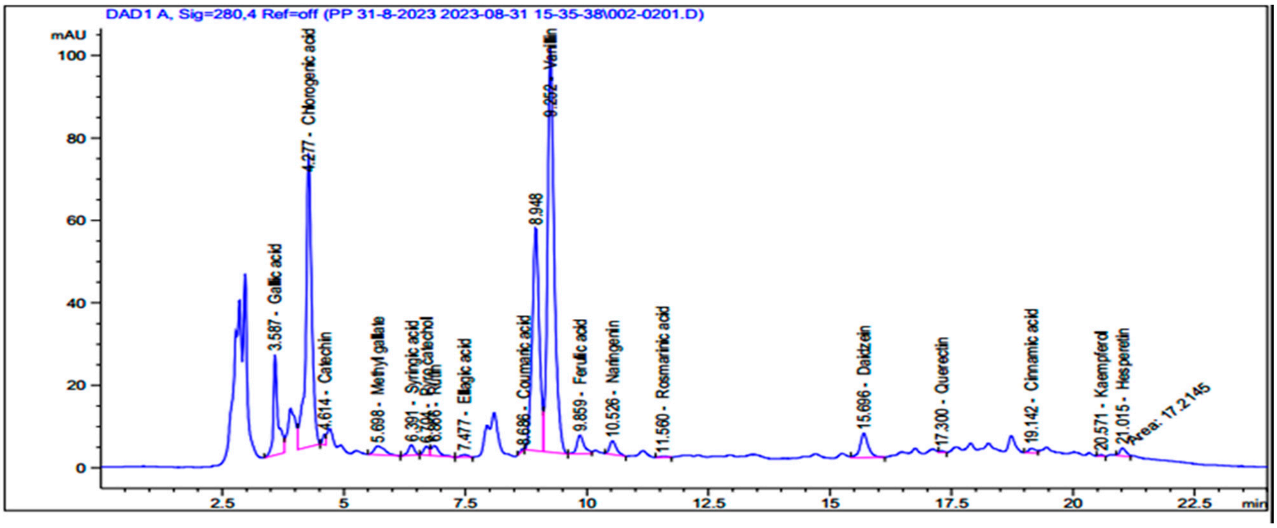

Figure S3. HPLC-MS analysis of methanol extract of *Calotropis procera.*
